# Diversity among Macroalgae-Consuming Fishes on Coral Reefs: A Transcontinental Comparison

**DOI:** 10.1371/journal.pone.0045543

**Published:** 2012-09-20

**Authors:** Adriana Vergés, Scott Bennett, David R. Bellwood

**Affiliations:** 1 Centre for Marine Bio-Innovation, Evolution and Ecology Research Centre, School of Biological Earth and Environmental Sciences, University of New South Wales, Sydney, New South Wales, Australia; 2 Sydney Institute of Marine Sciences, Mosman, Western Australia, Australia; 3 Centre for Marine Ecosystem Research, School of Natural Sciences, Edith Cowan University, Joondalup, Western Australia, Australia; 4 UWA Oceans Institute and School of Plant Biology, The University of Western Australia, Crawley, Western Australia, Australia; 5 Australian Research Council Centre of Excellence for Coral Reef Studies, School of Marine and Tropical Biology, James Cook University, Townsville, Queensland, Australia; Leibniz Center for Tropical Marine Ecology, Germany

## Abstract

Despite high diversity and abundance of nominally herbivorous fishes on coral reefs, recent studies indicate that only a small subset of taxa are capable of removing dominant macroalgae once these become established. This limited functional redundancy highlights the potential vulnerability of coral reefs to disturbance and stresses the need to assess the functional role of individual species of herbivores. However, our knowledge of species-specific patterns in macroalgal consumption is limited geographically, and there is a need to determine the extent to which patterns observed in specific reefs can be generalised at larger spatial scales. In this study, video cameras were used to quantify rates of macroalgae consumption by fishes in two coral reefs located at a similar latitude in opposite sides of Australia: the Keppel Islands in the Great Barrier Reef (eastern coast) and Ningaloo Reef (western coast). The community of nominally herbivorous fish was also characterised in both systems to determine whether potential differences in the species observed feeding on macroalgae were related to spatial dissimilarities in herbivore community composition. The total number of species observed biting on the dominant brown alga *Sargassum myriocystum* differed dramatically among the two systems, with 23 species feeding in Ningaloo, compared with just 8 in the Keppel Islands. Strong differences were also found in the species composition and total biomass of nominally herbivorous fish, which was an order of magnitude higher in Ningaloo. However, despite such marked differences in the diversity, biomass, and community composition of resident herbivorous fishes, *Sargassum* consumption was dominated by only four species in both systems, with *Naso unicornis* and *Kyphosus vaigiensis* consistently emerging as dominant feeders of macroalgae.

## Introduction

Herbivory is a key ecological process in coral reefs that supports intricate food webs and strongly contributes to the resilience of these systems, i.e. their ability to reorganise and maintain ecosystem function following disturbance [1], [2]. In recent decades, roving herbivorous fishes have been identified as key elements of coral reef communities and overfishing of these consumers is considered a significant factor contributing to reef degradation worldwide. This is often linked to phase shifts from coral to macroalgal dominance [3], [4], [5], [6]. However, roving herbivorous fishes do not constitute an ecologically uniform group, but rather comprise an agglomerate of species with widely varying feeding modes and diets [7], [8], [9], [10] that have been broadly categorised into grazer and browser functional groups [1], [11], [12]. The grazer functional group, which includes excavating and scraping species (primarily parrotfishes and acanthurids), is largely restricted to consuming algal turfs and the associated material in the epilithic algal matrix (EAM, sensu Wilson et al. [13]) and can therefore only limit macroalgal abundance by consuming recruits [1], [11]. In contrast, browsers are able to remove large erect macroalgae and thus have the potential to reverse phase shifts once macroalgae are established on reefs [14], [15].

An extensive body of literature from a wide range of coral reef systems shows that macroalgal browsers are highly selective, and that most species feed on a small subset of the available algal species [16], [17], [18], [19], [20]. Feeding selectivity has been linked to chemical and physical defences developed by many tropical algal species as a defence against herbivory [17], [18], [21]. In contrast, other tropical algal species that are highly susceptible to herbivory largely depend on spatial refuges to persist and are therefore only abundant in habitats characterised by low herbivore biomass or accessibility [22], [23], [24], [25], [26].

On the Great Barrier Reef (GBR), transplant experiments have shown that the abundance and distribution of *Sargassum* species are strongly influenced by herbivory [27]. Furthermore, herbivore-exclusion experiments have shown that this genus dominates macroalgal biomass in the absence of larger herbivorous fish, and can have catastrophic community-level effects because it depresses the fecundity, recruitment and survival of corals [28]. Despite *Sargassum* being considered susceptible to herbivory on the GBR, recent studies in this region have shown that removal of this macroalga is often dominated by only one or two browsing species [14], [29], [30], [31], [32]. This limited redundancy among consumers of macroalgae highlights the potential vulnerability of coral reefs to disturbance and stresses the need to assess the functional role of individual species of herbivores [32]. However, our knowledge of such species-specific patterns in macroalgal consumption is currently limited geographically, and there is a need to determine whether the patterns observed on specific reefs are applicable at a broader scale, especially beyond the GBR.

In this study, the rates of *Sargassum* consumption of individual fishes in the southern GBR (east coast of Australia) were directly compared with species-specific consumption patterns from a coral reef system located at similar latitude in the west coast of Australia, the Ningaloo Reef. Video cameras were used to quantify rates of macroalgal consumption by individual species and underwater censuses were performed to compare herbivorous fish communities in the two systems.

## Materials and Methods

### Study Locations

**Figure 1:**
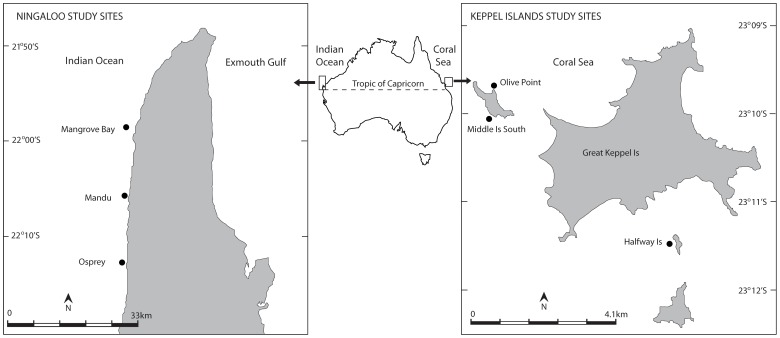
Map of the two study regions, the Keppel Islands in the southern Great Barrier Reef and Ningaloo Reef, showing the location of the study sites.

This study was conducted between December 2008 and February 2009 in the Keppel Islands Group (23° 109S, 151° 009E) on the GBR (East Australia) and on Ningaloo Reef (22° 07S, 113° 52E) in Western Australia (Figure 1). The Keppel Islands Group includes 15 islands located about 18 km from mainland Australia in the southern inshore GBR that are strongly influenced by the Fitzroy river catchment. The Ningaloo Reef is a fringing arid-zone reef approximately 290 km in length that forms a discontinuous barrier adjacent to the North West Cape, where expansive coral growth occurs within 100s of meters from the mainland.

Preliminary cross-habitat surveys were performed in the GBR and Ningaloo to identify analogous habitats within each system with similar coral-dominated benthic communities and relatively high levels of herbivorous fish biomass. At the Keppel Islands, the reef crest zone (∼3 m depth at high tide) was locally characterised by the highest coral cover (55.6±4.0%; mean ± SE) and the highest herbivore biomass across the fringing reef profile (137.59±28.64 kg ha^−1^; mean ± SE). In Ningaloo Reef the back-reef flat habitat (∼2 m depth at high tide), which is located a few meters inshore from the reef crest, supports the highest coral cover (40.7±3.7%; mean ± SE) and is characterised by the highest herbivore biomass across the fringing reef profile (1,865.27±604 kg ha^−1^; mean ± SE).

Within each region, three representative reefs (hereafter referred to as locations) were selected that were all situated within sanctuary zones, to minimise the potential effect of extractive activities. The three Keppel Island locations were Olive Point (23° 09S 150° 55E), Middle Island (23° 10S, 150° 55E) and Halfway Island.

(23° 11S, 150° 58E). The three Ningaloo locations were Mangrove Bay (21°58S, 113° 54E), Mandu (22°05S, 113° 52E), and Osprey (22°14S, 113° 52E). Within each location, two sites were haphazardly selected about 100 m apart.

### Macroalgal Assays and Video Analysis

Species-specific rates of consumption on the brown alga *Sargassum myriocystum* were measured in the Great Barrier Reef and in Ningaloo Reef using video cameras. The genus *Sargassum* was selected as a bioassay because it represents the most abundant algae in both coral reef systems [26], [27], [33]. *Sargassum myriocystum* J. Agardh was chosen because it is readily identifiable in the field and pilot studies indicated that it was palatable and readily eaten by fish within a few hours.

At each site, ten *S. myriocystum* assays (ca. 230 g) were haphazardly deployed on the reef. Five individual assays were tethered to the dead coral substratum using a rubber band and gardening wire, and five of the assays were protected from herbivores in cages (50×50×50 cm; 1.44 cm^2^ mesh size) to control for any biomass changes not due to herbivory by fish (e.g. handling losses and algal detachment due to water movement). Algae were deployed for approximately 4.5 hours between 8 am and 4 pm over three consecutive days. Fresh weight (to the nearest 0.1 g) was recorded before and after deployment. Algal biomass losses due to herbivory were calculated by randomly pairing individual treatment and control specimens [34] and subtracting the change in biomass of the treatment specimen from the change in biomass of the control specimen (uncaged – caged). Average biomass changes in control specimens were 10.15% of initial weight in the Keppels region and 18.66% of initial weight in Ningaloo (n = 90).

### Video Analysis

Two of the five treatment assays deployed at each site were filmed using a stationary underwater video camera (either a Sony DCR-HC1000E or a Sony HDR SR12 in an underwater housing) following the techniques of Hoey and Bellwood [32]. The three days at each site yielded approximately 81 hours of footage per region. The total number of bites per fish species and size (total length, TL) was recorded from the video footage for each sampling period. To account for variation in bite size related to differences in body size, the midpoint of each size class was used to calculate mass-standardised estimates of bite ‘impact’ for each fish species (total number of bites × body mass in kilograms) based on established length weight relationships from the literature (following Bellwood et al. [14]). Forays, where rapid consecutive bites by an individual fish took place without a discernable pause, were conservatively classed as a single bite [35].

Multivariate differences in the assemblages of fishes feeding on the *Sargassum myriocystum* bioassays were calculated using a three-way permutational analysis of variance (PERMANOVA) with the following factors: Region (2 levels, fixed), Location (3 levels, random, nested within Region), and Site (2 levels, random, nested within Location and Region). The Bray-Curtis distance was our metric in the multivariate analyses and data were fourth-root transformed prior to analyses to reduce the effects of numerically large values (i.e. abundant schooling species) [36]. Non-metric multidimensional scaling (nMDS) was used to produce two-dimensional ordinations of the similarities between multivariate fish samples. The similarity percentages procedure (SIMPER, [36]) was used to determine the fish taxa that characterised each region and contributed most strongly to dissimilarities between multivariate samples from the different regions. The contribution of each taxon was evaluated using the ratio of the mean overall dissimilarity between sets of samples and the standard deviation of this contribution (mδ_i_/SD[δ_i_]). Taxa were considered ‘important’ if this ratio was higher than 1 (i.e. the mean contribution was higher than the variation). All multivariate statistical analyses were performed using Primer-E v6 software [37] with the PERMANOVA+ add-on package (version 1.0.1 [38]).

### Relationship between Macroalgal Removal and Bite Rates of Individual Fishes

In order to identify the fish species that contributed most strongly to macroalgal removal in Ningaloo Reef and the Keppel Islands, the herbivorous fish species that were responsible for >5% of bites in each region were first selected (four species per region, see Results). Simultaneous multiple regression was then used to describe the relationship between algae removed in the filmed bioassays (dependent variable) and the corresponding mass-standardised feeding rates for that particular filmed replicate of the four herbivorous fish species and all other species pooled together (predictor variables; n = 36 filmed replicates per region; one analysis per region). Multiple regression analyses were performed using R software (Version 2.9.0 [39]).

### Distribution of Herbivorous Fishes

To identify whether potential differences in the species feeding on *Sargassum* were related to spatial dissimilarities in the fish community, roving herbivorous fish communities were censused at each region, location and site using standard underwater visual surveys. Fishes were counted on six replicate 10 minute timed swims per site during daylight hours by divers on SCUBA (avoiding 2 hours before and after sunrise) [40]. Fish counts were performed swimming at a constant speed and counting and estimating the size of fish within a 4 m wide transect (all censuses performed by SB). The length of each transect was subsequently measured using tapes (116±8.7 m mean ± SE). Fishes were identified to species level and their total length was estimated in 5 cm size categories. Density estimates were converted to biomass using the published allometric length-weight regressions [41]. Counts were restricted to fishes over 10 cm TL from the families Acanthuridae, Siganidae, Kyphosidae and Labridae (parrotfishes). Individuals belonging to the species *Acanthurus auranticavus, A. grammoptilus* and *A. blochii* were grouped as *Acanthurus* spp. due to difficulties in identification.

Multivariate differences between the fish assemblages counted in the underwater censes were calculated using a three-way PERMANOVA as described above. Two-dimensional ordinations of the similarities between multivariate fish samples were produced with nMDS plots, and SIMPER was used to determine the fish taxa that characterised each region and contributed most strongly to dissimilarities. Univariate differences in total herbivorous fish biomass were calculated with the same three-way factorial design using the statistical package GMAV [42]. Normality and equality of variances of the data were confirmed by visual inspection of scatterplots and distribution of residuals; Cochran’s test was further used to test equality of variances.

## Results

### Video Analysis

**Figure 2:**
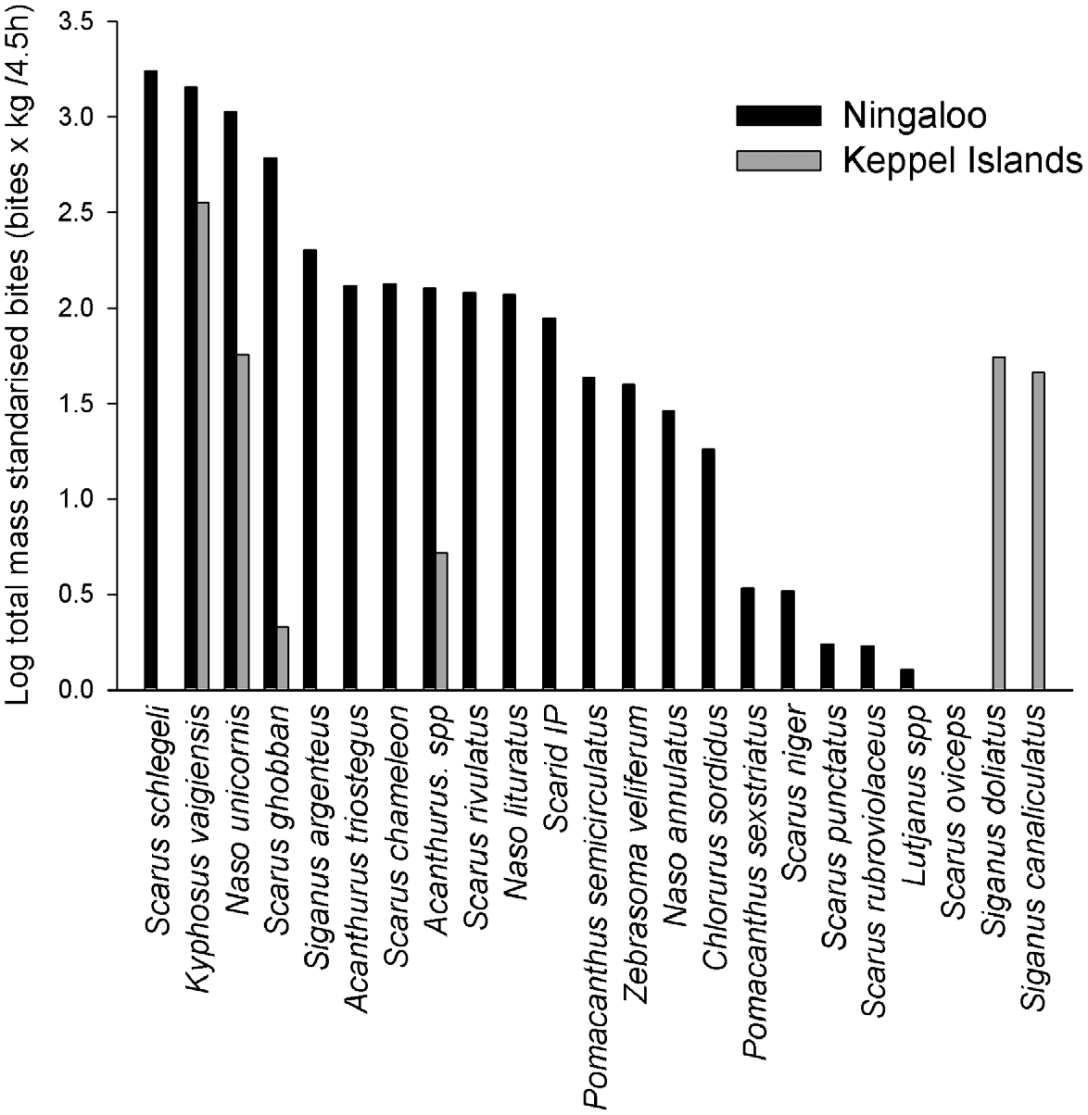
Total number of mass standardised bites (log transformed) taken by of the herbivorous fish assemblages feeding in each region over 4.5 hours (n = 6).

In Ningaloo Reef, 23 species of fish were recorded feeding on our *Sargassum* assays (Figure 2), which took a total of 15,792 bites. The following four species accounted for over 85% of all mass standardised bite rates: *Scarus schlegeli* (29.5%), *Kyphosus vaigiensis* (24.3%), *Naso unicornis* (18.0%) and *Scarus ghobban* (10.3%). A further 19 species accounted for the rest of the bites, with each species being responsible for <5% of mass standardised bites individually. In the Keppel Islands, only 8 species were observed feeding on our assays, which took a total of 1,085 bites. Four species were responsible for over 95% of all mass standardised bites in the Keppel Islands: *Kyphosus vaigiensis* (68.2%), *Naso unicornis* (10.9%), *Siganus doliatus* (10.5%) and *Siganus canaliculatus* (8.8%). The other Keppel Island fishes (four species) individually accounted for <5% of mass standardised bites.

**Figure 3:**
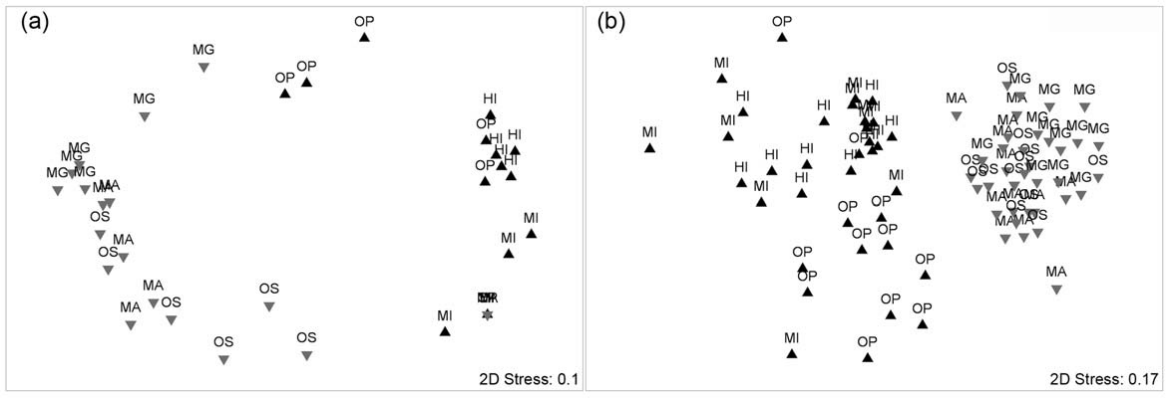
Non metric multidimensional scaling plots (nMDS) comparing: (a) the herbivorous fish assemblages feeding on the algal bioassays between regions (symbols) and locations (n = 6), and (b) the herbivorous fish assemblages surveyed using underwater visual census between regions (symbols) and locations (n = 6). All data were fourth-root transformed prior to ordination. Locations have been abbreviated as follows: HI = Halfway Island, MI = Middle Island, OP = Olive Point; MG = Mangrove Bay, MA = Mandu, OS = Osprey.

Strong differences in the mass standardised bite rates taken by the fish assemblages observed feeding on the macroalgal assays were recorded (Table 1 a), which were clearly separated on the nMDS ordination as two distinct groups (Figure 3a). Differences between the fish assemblages that fed in the different sites within each location were detected, but not between locations within the two regions (significant Site (Location (Region)) effect in Table 1 a). The SIMPER procedure identified two fish species that were characteristic of the Ningaloo assemblage of macroalgae-feeding fishes: *Scarus schlegeli* (mean similarity/standard deviation mδ_i_/SD[δ_i_] = 1.55) and *Scarus ghobban* (mδ_i_/SD[δ_i_] = 1.11). No species were identified as characteristic of the Keppels Islands region. The following six species contributed to the percentage dissimilarity between regions: *Scarus ghobban* (mδ_i_/SD[δ_i_] = 1.55); *Scarus schlegeli* (mδ_i_/SD[δ_i_] = 1.29), *Siganus argenteus* (mδ_i_/SD[δ_i_] = 1.25), *Acanthurus triostegus* (mδ_i_/SD[δ_i_] = 1.06), *Scarus rivulatus* (mδ_i_/SD[δ_i_] = 1.04), and *Naso unicornis* (mδ_i_/SD[δ_i_] = 1.02). Similar statistical results were obtained whether we analysed mass standardised bite rates (total number of bites × body mass in kilograms per 4.5 h) or bite rate data (total number of bites per species per 4.5 h; statistical results not shown).

**Table 1:** Results of the three factor analyses of variance assessing differences between regions, locations and sites in (a) Mass standardised bite rates from the fish community feeding on the algal bioassays (PERMANOVA, data fourth-root transformed), (b) Roving herbivorous fish community composition (PERMANOVA, data fourth-root transformed) and (c) Total roving herbivorous fish biomass (ANOVA).

	(a) Feeding fish community				(b) Fish community composition				(c) Total herbivorous fish biomass			
Source	df	MS	Pseudo-F	P	df	MS	Pseudo-F	P	df	MS	F	P
Region	1	34076	10.156	**0.002**	1	66643	11.855	**0.001**	1	85.958	13.57	**0.021**
Location (Reg)	4	3392.7	1.5847	0.152	4	5625	3.8345	**0.001**	4	6.334	4.93	**0.042**
Site (Loc (Reg))	6	2149.6	2.2401	**0.01**	6	1467.1	1.3954	0.057	6	1.286	0.89	0.505
Residual	22	959.58			59	1051.4			59	1.4370		

Significant probabilities are indicated in bold.

### Relationship between Bite Rates and Macroalgal Removal

In the Keppel Islands, the mass standardised bites of *Kyphosus vaigiensis, Naso unicornis, Siganus doliatus, S. canaliculatus* (the four species individually responsible for >5% bites) and all other species pooled, explained about 56% of the variation in the loss of algal biomass from our assays (F _5, 30_ = 7.6, p<0.001, adjusted R^2^ = 0.485; Table 2). However, partial regressions indicated that only the mass standardised bite rates of *Naso unicornis* at Keppel Islands had a significant effect on algal biomass loss of the filmed assays (Table 2). The relationship between macroalgal biomass loss in Ningaloo Reef and the mass standardised bite rates of *K. vaigiensis, N. unicornis, Scarus schlegeli, S. ghobban* and all other species pooled was marginally non-significant (F _5, 30_ = 2.18, p = 0.0833, adjusted R^2^ = 0.144).

**Table 2:** Results of multiple regression analysis on the relationship between algae biomass loss in the Keppel Islands and the standardised bite rate of the four species responsible for >5% of all bites and all other species pooled together.

Source	Estimate	Estimate SE	t	p
*N. unicornis*	95.225	36.172	2.633	**0.013**
*K. vaigiensis*	23.646	15.997	1.478	0.150
*S. doliatus*	8.251	20.090	0.411	0.684
*S. canaliculatus*	−6.100	19.627	−0.311	0.758
Sum all otherspecies	−15.785	46.709	−0.338	0.738

Overall model: Adjusted R^2^ = 0.48, F_5, 30_ = 7.6, p<0.001. Significant probabilities are indicated in bold.

### Distribution of Herbivorous Fishes

There were strong regional and location differences in the herbivorous fish community composition (Table 1 b). Regional differences were clearly displayed as two separate groups in the nMDS plot (Figure 3b). SIMPER analyses identified *Siganus doliatus* as the only species characteristic of the Keppel Islands (mδ_i_/SD[δ_i_] = 1.23). Five species characterised Ningaloo Reef samples: *Chlorurus sordidus* (mδ_i_/SD[δ_i_] = 3.64), *Acanthurus triostegus* (mδ_i_/SD[δ_i_] = 2.68), *Scarus schlegeli* (mδ_i_/SD[δ_i_] = 2.19), initial phase parrotfish (scarid IP; mδ_i_/SD[δ_i_] = 1.58), and *Scarus ghobban* (mδ_i_/SD[δ_i_] = 1.23). The following nine taxa contributed to the percentage dissimilarity between regions: *Chlorurus sordidus* (mδ_i_/SD[δ_i_] = 3.44); *Acanthurus triostegus* (mδ_i_/SD[δ_i_] = 2.41), *Scarus schlegeli* (mδ_i_/SD[δ_i_] = 1.92), *Siganus doliatus* (mδ_i_/SD[δ_i_] = 1.66), *Scarus ghobban* (mδ_i_/SD[δ_i_] = 1.46), *Scarus* IP (mδ_i_/SD[δ_i_] = 1.39), *Naso unicornis* (mδ_i_/SD[δ_i_] = 1.3), *Scarus rivulatus* (mδ_i_/SD[δ_i_] = 1.29), and *Acanthurus* sp. (mδ_i_/SD[δ_i_] = 1.17).

**Figure 4:**
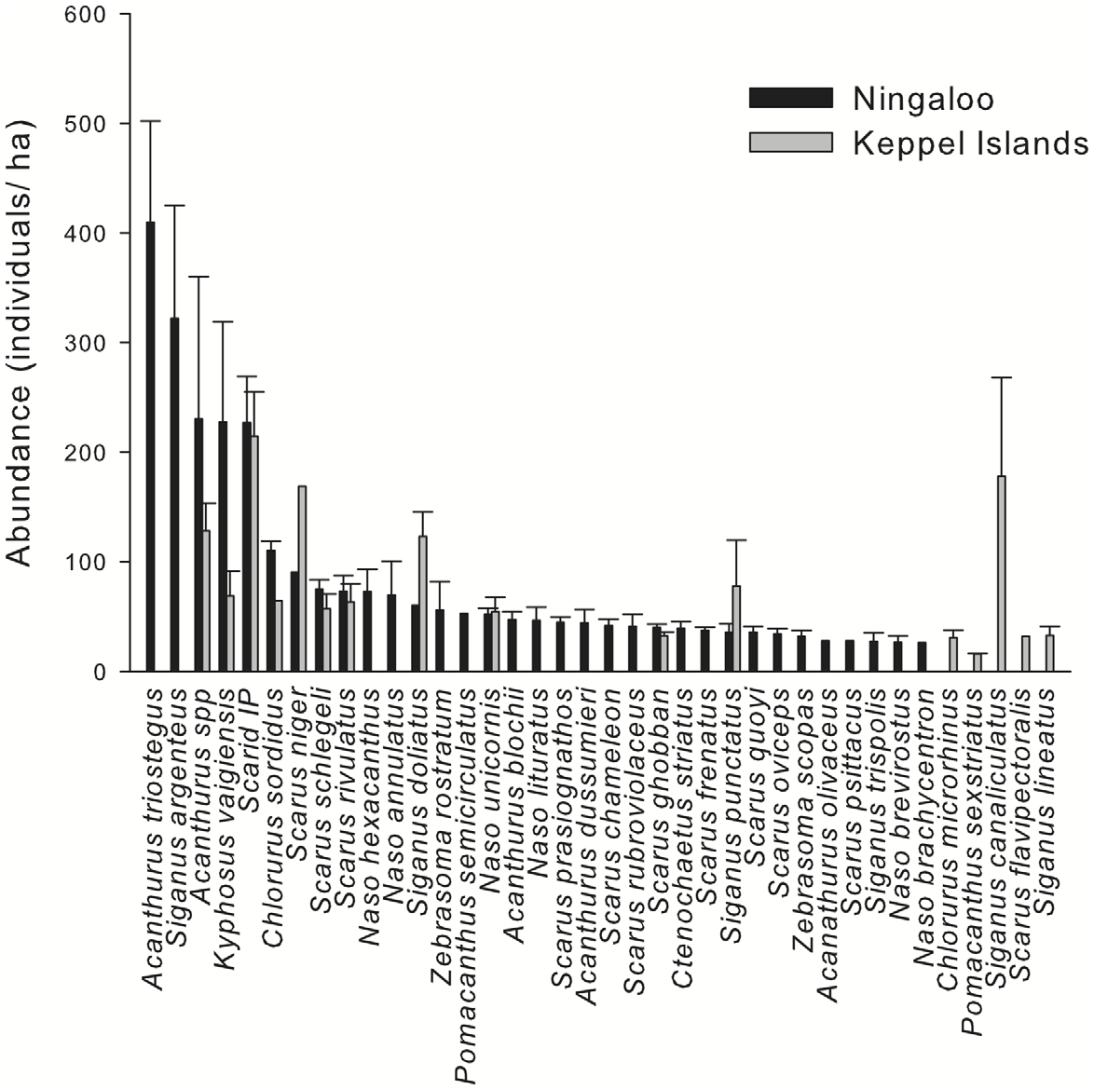
Abundance of herbivorous fish species surveyed using underwater visual censuses at each region (n = 6).

**Figure 5:**
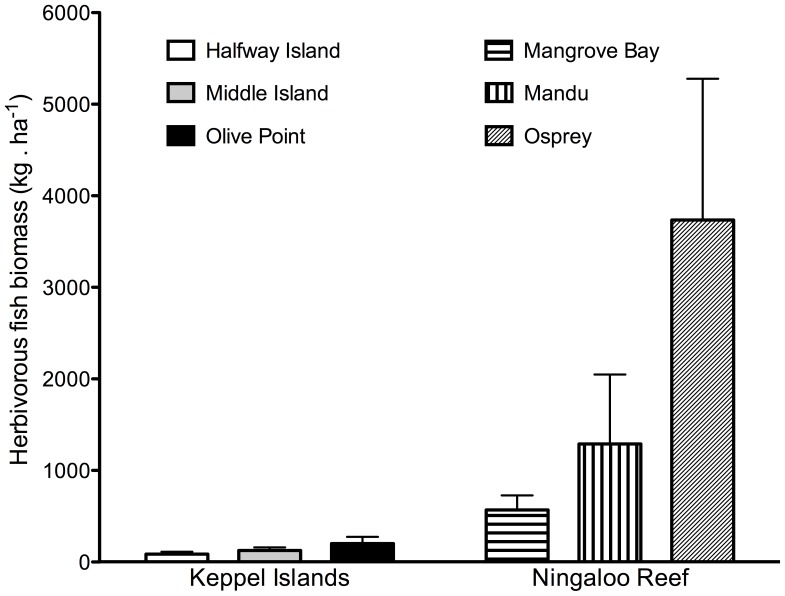
Variation between regions and locations in total roving herbivorous fish biomass (n = 6). Sites were not significantly different from each other and have been pooled.

There were striking differences in species diversity, with 33 species being censused in Ningaloo Reef compared with only 16 in the Keppel Islands (Figure 4). Similarly, there were significant regional differences in total biomass of all roving herbivorous fish, with Ningaloo Reef biomass values being over 13 times those of the Keppel Islands (Figure 5; Table 1 c). In Ningaloo Reef, there were differences in total fish biomass between locations (SNK post-hoc tests), but not between sites in any of the two regions (Table 1 c).

## Discussion

In this study, a small number of fish species were observed doing the bulk of the feeding on macroalgae in two geographically distant Indo-Pacific coral reefs. Despite strong differences between Ningaloo Reef and the Keppel Islands in both the total diversity of species observed feeding on *Sargassum* and in the species composition of the roving herbivorous fish communities, four species were responsible for over 80% of all bites in both systems. The diversity of fish species recorded biting the algal bioassays in Ningaloo Reef (23 species) was much higher than in the Keppel Islands (8 species) and is one of the highest recorded on coral reefs to date (cf. 20 species in the northern GBR [29]).

There were striking differences in the species composition and total biomass of roving herbivorous fish between the two regions. Ningaloo Reef hosted a diverse assemblage of roving herbivores, with biomass values that were an order of magnitude higher than in the comparatively depauperate fish assemblages of the Keppel Islands. Differences in herbivorous fish communities of a similar magnitude are also observed across different regions of the GBR, with inshore reefs having significantly lower abundance and diversity of roving herbivores than mid-shelf and outer-shelf reefs [33], [43], [44]. Thus, despite Ningaloo Reef being found in close proximity to the mainland (within meters), its roving herbivorous fish community is more comparable in magnitude to mid-shelf and outer-shelf reefs in the GBR than to inshore reefs such as the Keppel Islands [26], [45]. This may be related to several physical conditions that strongly limit the influence of the human disturbances and the mainland on Ningaloo Reef. This western coast reef is located in an arid zone where evaporation rates far exceed annual rainfall, hence minimising terrestrial run-off and its effect on turbidity and sediment load [46]. Additionally, anthropogenic impacts are extremely low in Ningaloo Reef, with low human populations, no agricultural activities, and limited commercial fishing activity. In contrast, inshore GBR reefs are strongly influenced by nutrient and pesticide loads and increasing sediment from several degraded river catchments due to agricultural activities and other land-use practices [47], [48], [49]. Despite these strong differences in history, species richness and community composition, the two areas exhibited a remarkably similar functional capacity. In both locations herbivory on macroalgae was restricted to a few, mostly shared, species.

In previous studies that have aimed to identify the key fish species responsible for consumption of *Sargassum* in the Great Barrier Reef, four different species have been identified as important (*Naso unicornis*, *Kyphosus vaigiensis*, *Siganus canaliculatus*, and *Platax pinnatus*), and a common pattern has emerged whereby a single species has dominated feeding at the local level [14], [30], [31], [32], [50], [51]. However, most previous studies were performed in the central to northern regions of the GBR. Our Keppel Islands results provide further confirmation of this pattern by highlighting the key role of one species, *N. unicornis*, in removing *Sargassum* in southern GBR inshore reefs, and suggest that at least some of these species are equally important in other Indo-Pacific reefs.

Although there were significant differences in the fish assemblages observed feeding in the two coral reef systems, *Kyphosus vaigiensis* and *Naso unicornis* were responsible for some of the highest mass standardised bite rates in both Ningaloo and the Keppel Islands, and have been recognised as important macroalgal consumers in other studies [30], [32], [52]. Our Ningaloo results are broadly consistent with a recent study performed across 300 km in this coral reef system, which shows that *N. unicornis* and *Kyphosus* spp. are key algal browsers in Ningaloo [53]. Although kyphosids are a minor component of fisheries, *Naso unicornis* is a heavily fished species throughout most of its range [32]. As such, a key component of a critical functional group may be at significant risk in many reef ecosystems.

Our results therefore confirm the key role of some species identified as important in previous studies (*Naso unicornis* and *Kyphosus vaigiensis*). However, the large differences in the number of species observed feeding on *Sargassum* in the two regions suggest that the Keppel Islands may have limited resilience when compared to other reefs such as Ningaloo, where functional redundancy among macroalgal consumers appears to be somewhat broader. This would be consistent with recent experimental evidence that shows that higher diversity of herbivorous fish can significantly lower macroalgal abundance in coral reefs [20], [54], and with studies that integrate long-term data sets of field surveys in the GBR which point to a strong association between low fish herbivore diversities and a coral-macroalgal phase-shift [3]. Nevertheless, while there was a large number of species observed feeding on the *Sargassum* bioassays in Ningaloo, it is not clear that these fishes were targeting macroalgae *per se*. Our analyses only detected a marginally non-significant relationship between the number of bites of the main consumers (*Naso unicornis*, *Kyphosus vaigiensis, Scarus schlegeli and S. ghobban*) and algal biomass removed. This was probably due to the high number of bites taken by the scarids that characterised feeding in Ningaloo. *S. schlegeli* and *S. ghobban* were observed taking many small bites and appeared to be feeding on epibiota and/or on surface detritus, i.e. not on the macroalgae thallus itself. This kind of feeding behaviour has been identified in other scarids (e.g. *Scarus rivulatus* on the GBR [52]), and both *S. schlegeli* and *S. ghobban* have been identified as scrapers (i.e. consumers of EAM) in studies based on their jaw morphology and field observations [35].

Overall, our results show that despite vast dissimilarities in the geomorphology of two widely spaced coral reef systems, and despite important differences in the diversity, biomass and community composition of resident herbivorous fishes, a small number of herbivorous fish species are critically important for the removal of established macroalgae on coral reefs. These findings therefore support the call for conservation programs [32] that focus on the maintenance of algal removal as a key ecological process requiring the protection of functionally dominant species.
